# Notch Signal Mediates the Cross-Interaction between M2 Muscarinic Acetylcholine Receptor and Neuregulin/ErbB Pathway: Effects on Schwann Cell Proliferation

**DOI:** 10.3390/biom12020239

**Published:** 2022-02-01

**Authors:** Roberta Piovesana, Annalinda Pisano, Simona Loreti, Ruggero Ricordy, Claudio Talora, Ada Maria Tata

**Affiliations:** 1 Department of Biology and Biotechnology Charles Darwin, Sapienza University of Rome, 00185 Rome, Italy; roberta.piovesana@umontreal.ca (R.P.); annalinda.pisano@uniroma1.it (A.P.); simona.loreti@uniroma1.it (S.L.); 2 Department of Radiological, Oncological and Pathological Sciences, Sapienza University of Rome and Policlinico Umberto I, 00185 Rome, Italy; 3 Institute of Molecular Biology and Pathology, CNR, 00185 Rome, Italy; ruggero.ricordy@gmail.com; 4 Department of Molecular Medicine, Sapienza University of Rome, 00185 Rome, Italy; claudio.talora@uniroma1.it; 5 Research Centre of Neurobiology Daniel Bovet, 00185 Rome, Italy

**Keywords:** Schwann cells, M2 muscarinic receptor, Notch-1, Neuregulin-1, erbB receptors, cell proliferation

## Abstract

The cross-talk between axon and glial cells during development and in adulthood is mediated by several molecules. Among them are neurotransmitters and their receptors, which are involved in the control of myelinating and non-myelinating glial cell development and physiology. Our previous studies largely demonstrate the functional expression of cholinergic muscarinic receptors in Schwann cells. In particular, the M2 muscarinic receptor subtype, the most abundant cholinergic receptor expressed in Schwann cells, inhibits cell proliferation downregulating proteins expressed in the immature phenotype and triggers promyelinating differentiation genes. In this study, we analysed the in vitro modulation of the Neuregulin-1 (NRG1)/erbB pathway, mediated by the M2 receptor activation, through the selective agonist arecaidine propargyl ester (APE). M2 agonist treatment significantly downregulates NRG1 and erbB receptors expression, both at transcriptional and protein level, and causes the internalization and intracellular accumulation of the erbB2 receptor. Additionally, starting from our previous results concerning the negative modulation of Notch-active fragment NICD by M2 receptor activation, in this work, we clearly demonstrate that the M2 receptor subtype inhibits erbB2 receptors by Notch-1/NICD downregulation. Our data, together with our previous results, demonstrate the existence of a cross-interaction between the M2 receptor and NRG1/erbB pathway-Notch1 mediated, and that it is responsible for the modulation of Schwann cell proliferation/differentiation.

## 1. Introduction

In recent decades, several studies have highlighted the importance of neuron-glia cross-talk in the regulation of axonal conduction, synaptic transmission and glial differentiation [1,2,3]. The bidirectional communication between Schwann cells (SCs) and axons is essential during development, but also in the postnatal period and adult life. The extrinsic signals inducing SC proliferation/differentiation are only partially known [4]. Neuregulins (NRGs) are one of the crucial axons-derived growth factors that drive SC development and differentiation. The NRGs family is composed of four members (NRG-1, -2, -3, -4) encoded by four distinct genes [5]. They bind with different affinity the epidermal growth factor receptors, erbB [6]. NRG1, the most characterized NRG member, can be produced in three different isoforms (types I, II, and III) derived by alternative splicing from the same gene. It can be produced as soluble membrane-bound protein that can be released as paracrine factor after being cleaved by proteases, or remain on the cell surface where it mediates juxtacrine interactions [7]. Because different NRG1 isoforms can be produced and many of these can be also released, the erbB receptors may receive this paracrine signal directly by neighbouring cells. However, occasionally, the same cell can produce and receive the NRG signal in an autocrine manner.

NRG1/erbB signalling plays a critical role during SC development [5]. NRG1 type I is expressed on the peripheral nervous system (PNS) and it has been indicated as a key molecule in modulating SC survival, proliferation, and to drive the SC plasticity after peripheral nerve injury [7].

Depending on the developmental stage, NRG1/erbB pathway significantly regulates different SC physiological processes (i.e., survival, proliferation, migration, differentiation and myelination) [5,6,8,9]. This functional variety could be dependent on the NRG type and by the interaction of NRG1/ErbB signalling with other pathways.

NRG1 type I has been shown to regulate Schwann cell plasticity and remyelination after peripheral nerve injury [7]. Axonally derived NRG1 type III is an essential modulator of Schwann cells during development, axonal sorting, and remyelination [8]. In addition, NRG1 type III is known to inhibit NRG1 type I expression in SCs (via ErbB2 and MAPK). NRG1 type I is highly expressed after injury in the absence of axon-glia contact [10].

Neurotransmitters and their receptors mediate electrical neuronal signalling, but it has been demonstrated that they also play a fundamental role as signalling molecules for the survival and differentiation of both neurons and glial cells [11,12,13,14,15]. In particular, acetylcholine (ACh) regulates glial cell proliferation and differentiation through the expression of different cholinergic receptors in oligodendrocytes (OLs), astrocytes and SCs [16,17,18,19]. Our previous data showed that SCs express different muscarinic receptors with a major expression of M2 subtype [16]. ACh via M2 receptor, decreasing intracellular cyclic AMP (cAMP) levels, negatively modulates SC proliferation, causing a reversible arrest of cell cycle with cell accumulation in G1 phase [20]. Moreover, the selective stimulation of M2 subtype through the preferential agonist arecaidine propargyl ester (APE), modulates NGF production and maturation in SCs, decreasing proNGF-B (25kDa) isoform, involved in apoptotic processes [21,22]. Interestingly, the M2 receptor subtype is also expressed by human SCs [23] and, similarly to that observed in rats, the activation of M2 receptors inhibits SC proliferation and upregulates the differentiation factor Egr2/Krox20 also in human SCs [23]. Starting from these previous data and considering the role of the NRG pathway in SC development, the aim of this work was to investigate the possible cross-interaction between the M2 receptor and the NRG/ErbB pathway.

## 2. Materials and Methods

### 2.1. Statements for Experiments Involving the Use of Animals

All the experiments requiring animals were performed following terminal anaesthesia with CO_2_ and cervical dislocation, in accordance with the protocol (7FF2C.6.EXT. 96), approved by the Ministry of Health (Aut. N. 1184/2016-PR 16/12/2016 to A.M.T.). Sciatic nerves were collected from 2-day-old Wistar pups and processed as indicated in the next paragraph.

### 2.2. Schwann Cell Cultures

Sciatic nerves were collected from 2-day-old Wistar pups, according to the protocol modified by Davis and Stroobant [24]. Briefly, nerves were digested with collagenase Type I (Sigma-Aldrich, St. Louis, MA, USA) and 2.5% trypsin (*v*/*v*, Sigma-Aldrich, St. Louis, MA, USA). SCs were seeded onto Poly-L-Lysine-coated 75 cm^2^ flasks with high-glucose Dulbecco’s Modified Eagle’s Medium (DMEM, Sigma-Aldrich, Milan, Italy) containing 10% fetal bovine serum (FBS, Sigma-Aldrich, Milan, Italy). For the depletion of fibroblasts, cells were treated with 1 mM cytosine arabinoside (AraC, Sigma-Aldrich, Milan, Italy) for 48 h and then with anti-Thy 1.1 (1:1000, Serotec, Bio-Rad group, Hercules, CA, USA) and rabbit complement (1:2 *v*/*v*; Cedarlane, Burlington, ON, Canada). SCs were then amplified in high-glucose DMEM, 10% FBS, 5 µM forskolin (Fsk; Sigma-Aldrich, Milan, Italy) and bovine pituitary extract [PE, 1:150 (*v*/*v*), Sigma-Aldrich, Milan, Italy]. For the following experiments, cells were maintained in DMEM, 10% FBS and 2 µM Fsk in T75 flasks at 37 °C with 10% CO_2_.

### 2.3. Drug Treatments

Arecaidine Propargyl Ester hydrobromide (APE, Sigma-Aldrich, Milan, Italy) is a preferred agonist of the M2 muscarinic receptor subtype. Its selectivity has been previously determined by pharmacological binding experiments and M2 knockdown in different cell models [20,25,26,27]. As previously used in other works, APE was used at the final concentration of 100 μM [20,25,28,29]. Pituitary extract (PE, Sigma-Aldrich, Milan, Italy) is derived from the pituitary gland and contains several hormones including glial growth factor (GGF); it was used at a final dilution of 6 µL/mL. NRG1 (Immunological Sciences, Rome, Italy) was used at the final concentration of 50 ng/mL. NRG1 or PE were added 1 h before APE treatment.

All experiments were performed in technical and experimental triplicate.

### 2.4. Cell Viability

SCs were seeded on a 24-well plate at a density of 50 × 10^3^ cells/well. The day after, cells were treated as described in “drug treatments”; in the co-treatment, NRG was added 1 h before APE. Cell growth was assessed by colorimetric assay based on 3-(4,5-dimethylthiazol 2-y1)-2,5-diphenyltetrazolium bromide (MTT; Sigma–Aldrich, Milan, Italy). For each well, the optical density (OD) at 570 nm was measured by the GloMax Multi Detection System (Promega, Milan, Italy).

### 2.5. Flow Cytometry Analysis

SCs were treated as described in “Drug treatments”, for 16, 24 and 48 h. At the end of the treatment, cells were incubated for 90 min with bromodeoxyuridine (BrdU, Sigma–Aldrich, Milan, Italy) at a final concentration of 45 μM, collected by trypsinization, centrifuged for 10 min at 1,000 rpm and washed three times with PBS. Cells were then fixed in methanol/PBS (1:1; *v*/*v*). To identify cells in S phase, DNA content and BrdU incorporation were determined in simultaneous analysis by staining with propidium iodide (PI) and anti-BrdU, respectively. Partial DNA denaturation was performed by incubating the cells in 3 N HCl for 45 min, followed by neutralization with 0.1 M sodium tetraborate.

Samples were then incubated with monoclonal anti-BrdU (1:50 *v*/*v*; Dako, Santa Clara, CA, USA) for a further 30 min at room temperature (RT), washed twice with 0.5% Tween-20 in PBS and incubated for 30 min with anti-mouse Alexa-488-fluor-conjugated (1:600; Promega, Milan, Italy). Samples were washed twice with 0.5% Tween-20 in PBS and finally stained with 10 μg/mL PI for 15 min at RT.

Similarly, for the analysis of erbB3 positive cells, SCs were cultured in the absence or in the presence of PE [PE, 1:150 (*v*/*v*), Sigma-Aldrich, Milan, Italy] and APE 100 µM for 24 h. Then, the cells were collected and fixed as reported above and incubated with rabbit polyclonal anti-ErB3 (C17) (1:300, Santa Cruz, sc-285, Dallas, TX, USA), followed by washing with 0.5% Tween-20 in PBS and an incubation of 30 min with anti-rabbit Alexa-488-fluor-conjugated (1:600; Promega, Milan, Italy).

Flow cytometry analysis was performed with a flow cytometer Coulter Epics XL with 488 nm wavelength excitation and 10^4^ events were collected for each sample. Biparametric (DNA content versus BrdU content) analysis was performed using WinMDI 2.7 software.

### 2.6. RT-PCR and qRT-PCR Analysis

Total RNA was extracted using Tri-Reagent (Sigma–Aldrich, Milan, Italy) and digested with DNAseI (Ambion–Life technologies Italia, Monza, Italy). Total RNA was reverse-transcribed into cDNA with 1 μg of random Primers (Promega, Milan, Italy) and 200 U of Moloney Murine Leukaemia Virus (M-MLV reverse) transcriptase (Promega, Milan, Italy). Glyceraldehyde-3-phosphate dehydrogenase (Gapdh) was used as the housekeeping gene. qRT-PCR was performed with SYBR Green Mastermix (Promega, Milan, Italy) and specific primers at a final concentration of 200 nM were added at the respective wells and analysed by Thermofisher Quantstudio3 (Waltham, MA, USA). Data were normalized for the housekeeping gene gapdh and the ΔΔCt method was used to determine the fold changes in the gene expression compared with the control.

The sequences of the primers used were:

*Nrg1* Forward 5′-CCATCACTCCACGACTGTC-3′
Reverse 5′-GTGCCTGCTGTTCTCTACC-3′
*Nrg1/I* Forward 5′-TCATCTTCGGCGAGATGTCTG-3′
Reverse 5′-CTCCTGGCTTTCATTTCTTTCA-3′
*erbB2* Forward 5′-CGAGTGTCAGCCTCAAAACA-3′
Reverse 5′-CTCATCCGGGTACTTCCAGA-3′
*erbB3* Forward 5′-CTGTTTAGGCCAAGCAGAGG-3′
Reverse 5′-GACTTTGTTTGCCTTCTCGC-3′
*Gapdh* Forward 5′-GTGCCAGCCTCGTCTCATAG-3′
Reverse 5′-TGATGGCAACAATGTCCACT-3′

### 2.7. Protein Extraction and Western Blot

Protein samples were extracted in a Lysis buffer (10 nM Tris, 0.5% NP40, 150 mM NaCl). A sample buffer (4×) was added to the protein samples, and they were heated for 5 min at 100 °C, loaded onto 10% SDS (Sodium dodecyl sulphate) polyacrylamide gel and run at 30 mA using a running buffer (25 mM Tris, 190 mM glycine, 0.08% [*w*/*v*] SDS). SDS-PAGE gels were transferred for 2 h onto PVDF membranes (Millipore, Billerica, MA) at 200 mA in a transfer buffer (20 mM Tris; 150 mM glycine, 10% [*v*/*v*] methanol). After transfer, membranes were blocked for 1 h in a blocking buffer [Tris-buffer saline (TBS)-Tween solution containing 5% non-fat dry milk (Cell Signalling Technology, Davers, MA, USA)]. Membranes were incubated with the primary antibodies [anti-HER2/ErbB2 (C-18), 1:800, Santa Cruz, Dallas, TX, USA); anti-ErB3 (C17) (1:800, Santa Cruz, Dallas, TX, USA)], previously diluted in the blocking solution, overnight at 4 °C. The day after, membranes were incubated with HPR-conjugated secondary antibody (1:20,000, Promega, Milan, Italy). Protein expression was detected by using enhanced chemiluminescence (ECL, Euroclone, Pero, Italy). β-actin or Calreticulin were used as protein reference [anti-β-actin, 1:2000, (Immunological Sciences, Rome, Italy); anti-calreticulin, 1:2000, (Abcam, Cambridge, UK)].

To detect β-actin, anti-mouse IgG alkaline phosphatase-conjugated secondary antibody was used. Bands were stained with nitro blue tetrazolium in the presence of 5-bromo-4-chloro-3-indolyl-phosphate (NBT-BCIP).

The optical density (OD) of each protein band was analysed with ImageJ software (National Institutes of Health, NIH, 469 Bethesda, MD, USA) and normalized against the OD of the protein reference band.

### 2.8. Immunocytochemistry

SCs were plated onto 35 mm diameter dishes in complete medium (DMEM + 10% FBS + 2 µM forskolin). Cells were washed twice with PBS and fixed for 20 min in 4% paraformaldehyde (PFA, Sigma-Aldrich, Milan, Italy) in PBS, at RT. After three washes in PBS, SCs were incubated for 45 min in 0.1% Triton X-100 (Sigma-Aldrich, Milan, Italy), 10% normal goat serum (NGS, Vector Laboratories, Burlingame, CA, USA) and 1% bovine serum albumin (BSA; Sigma-Aldrich, Milan, Italy) in PBS. Then, the cells were incubated overnight at 4 °C with the following primary antibodies: rabbit polyclonal anti-Neuregulin-1 (H210)(1:200, Santa Cruz, sc-28916, Dallas, TX, USA); rabbit polyclonal anti-HER2/ErbB2 (C18), (1:300, Santa Cruz sc-284, Dallas, TX, USA); mouse polyclonal anti-GM130, (1:300, Abcam, ab169276, Cambridge, UK); mouse monoclonal anti-Lamp1 (H4A3), (1:200, Abcam, ab25630, Cambridge, UK); rabbit polyclonal anti-GRP78 BIP, (1:300, Abcam, ab53068, Cambridge, UK), diluted in 0.1% Triton X-100, 1% NGS, 1% BSA in PBS. After three washes in PBS, SCs were incubated for 1 h at RT with the appropriate secondary antibodies: goat anti-rabbit or mouse IgG- Alexa 488 or 594-conjugated (Promega, Milan, Italy), diluted 1:500 in PBS + 0.1% Triton X–100 + 1% NGS. After three washes in PBS, the slides were mounted with Vectashield (H1200, Vector Lab, DBA, Milan, Italy).

### 2.9. Cell Infection with Adenovirus Expressing Notch-NICD

SCs were infected with recombinant adenoviruses expressing the constitutively active form of Notch-1 (NICD) and green fluorescent protein (GFP), using previously established protocol [30]. Viruses were used at a multiplicity of infection of 50 MOI. SCs were infected with adeno-GFP or adeno-GFP-NICD for 1 h in a serum free medium. After 1 h, complete media were replaced. The day after, cells were treated with APE for 24 h and then SCs were collected for Western blot analysis.

### 2.10. Data Analysis

Data analyses were performed with GraphPad Prism 8 (Graphpad Software, La Jolla, CA, USA). Data were presented as the average ± standard error of the mean (SEM). Student’s t-test or one-way ANOVA analyses with Bonferroni’s post-tests were used. A value of *p* < 0.05 was considered statistically significant: * *p* < 0.05, ** *p* < 0.01, *** *p* < 0.001 and **** *p* < 0.0001. The densitometric analyses of Western blot and PCR bands were measured by ImageJ software (National Institutes of Health, NIH, 469 Bethesda, MD, USA).

## 3. Results

### 3.1. M2 Receptor Stimulation Counteracts SC Proliferation Mediated by PE/NRG1

Previous data have demonstrated that M2 agonist APE was able to arrest rat and human SC proliferation [20,23]. In order to explain the mechanism responsible for this effect, we firstly evaluated the ability of M2 agonist APE to counteract SC proliferation induced by PE/NRG1. PE contains several factors including glial growth factors (GGF/NRG), for this reason it has been largely used in SC cultures to promote cell proliferation [20].

**Figure 1:**
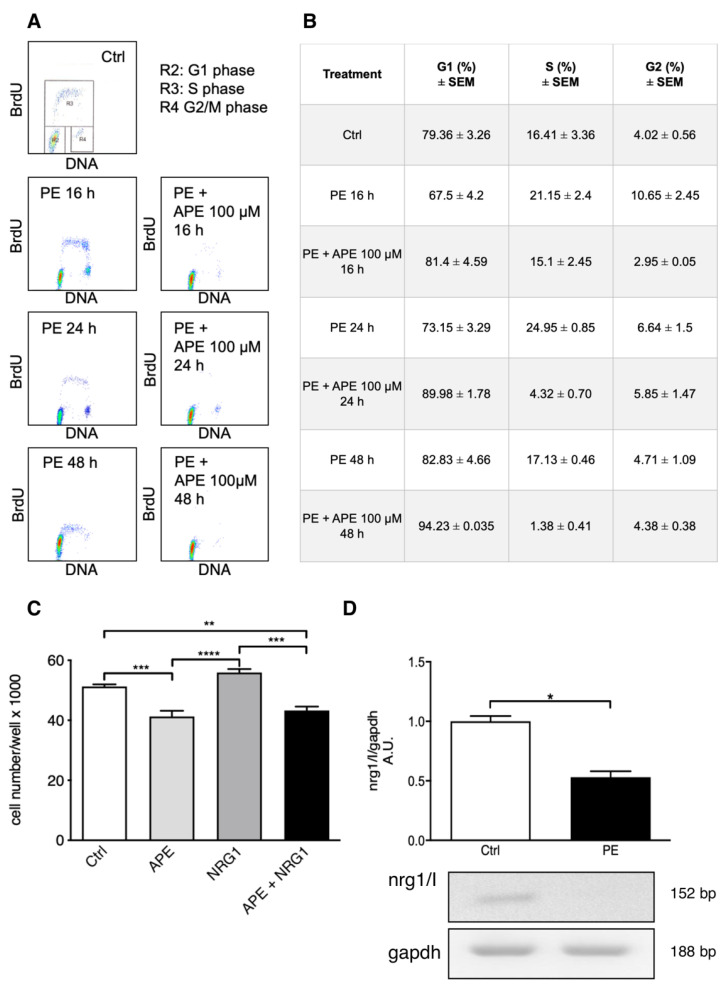
M2 selective stimulation counteracts SC proliferation-PE/NRG-mediated. (**A**,**B**) Untreated SCs present the typical proliferating profile with a percentage of cells in S phase 16.41 ± 3.36% (*n* = 3). Cells treated with PE show an increase in S phase (16 h: 21.5 ± 2.4%; 24 h: 24.95 ± 0.85%; 48 h: 17.13 ± 0.46%; *n* = 3). APE co-treatment is able to counteract the cell proliferation PE-induced, with a strong reduction in cells in S phase (R3 region, panel A). (**B**) The percentage of S phase decreases progressively with the increase in time of APE exposure in PE treated cells (16 h: 15,1 ± 2,45%; 24 h: 4,32 ± 0.70%; 48 h: 1,38 ± 0,41%; *n* = 3). (**C**) MTT assay shows that 48 h of APE treatment decreases SC growth (Ctrl vs. APE, *** *p* < 0.001; *n* = 3), whereas NRG1 exposure increases cell number (APE vs. NRG1, **** *p* < 0.0001; *n* = 3). APE plus NRG1 co-treatment shows a cell growth comparable to APE treatment (NRG1 vs. APE + NRG1, *** *p* < 0.001; Ctrl vs. APE+NRG1, ** *p* < 0.01; *n* = 3). (**D**) Representative RT-PCR shows a significant decrease in the transcript levels of nrg1 type I in SCs after 24 h of PE treatment. Gapdh was used as housekeeping gene. The graph reports the average of the OD ± SEM of the bands normalized against the gapdh of three independent experiments (Ctrl vs. PE, * *p* < 0.05; *n* = 3).

Cytofluorimetric analysis confirmed that PE treatment increased the percentage of the SCs in S phase already after 16 h of treatment (Figure 1A,B). In fact, SCs maintained in FBS and forskolin, showed the typical cell proliferating profile, with a percentage of S phase of 16.41% ± 3.36%, whereas, after PE exposure, the percentages were 21.15 ± 2.4% after 16 h, 24.95 ± 0.85% after 24 h and 17.13 ± 0.46% after 48 h, supporting the idea that PE has a positive effect on SC proliferation. This increase was counteracted by APE treatment already after 16 h of treatment. After 24 h and 48 h, the M2-mediated decrease of the cells in S phase was more evident (Region R3, Figure 1A). In fact, after 16 h of co-treatment, the percentage of cells in S phase was 15.1 ± 2.45% but, after 48 h, it was strongly reduced to 1.38 ± 0.41%. In order to understand if the effect of PE may be explained by the presence of GGF/NRG1, MTT assays were performed upon NRG1 and APE treatments (Figure 1C). As already demonstrated, NRG1 treatment increased cell growth, whereas APE treatment reduced significantly the cell growth; interestingly, APE plus NRG1 co-treatment showed the same cell number observed after APE treatment, confirming the inhibitory effect of APE on cell proliferation-NRG1 induced. It was also interesting that SCs treated with PE showed a significant downregulation of the transcript levels of nrg1 type I (Figure 1D), suggesting that NRG1 present in PE negatively counteracted its autocrine production by SCs.

### 3.2. M2 Receptor Activation Downregulates NRG1 Expression

**Figure 2:**
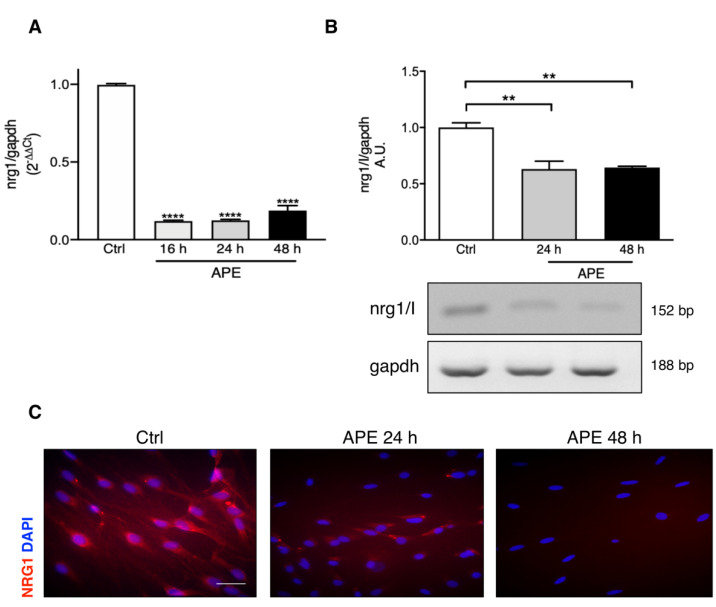
NRG1 transcript and protein levels are modulated by M2 agonist APE. (**A**) M2 receptor agonist APE downregulates *nrg1* transcript levels already after 16 h of treatment, up to 48 h of exposure (APE 16 h, 24 h, 48 h vs. Ctrl: **** *p* < 0.0001; *n* = 3) (**B**) RT-PCR shows SCs express the *nrg1 type I* isoform; M2 selective activation significantly downregulates this isoform after 24 h and 48 h of APE treatment (APE 24 h, 48 h: ** *p* < 0.01, *n* = 3). (**C**) Immunostaining shows a gradual reduction in NRG1 protein expression in SCs treated with the M2 agonist APE, until it disappears completely after 48 h of exposure (scale bar = 100 μm).

M2 activation, APE-mediated, significantly downregulated nrg1 transcript levels already after 16 h of APE exposure (Figure 2A). Using different primers able to recognize the nrg1 type I isoform, RT-PCR analysis showed a significant downregulation of the nrg1/I transcripts after 24 h and 48 h of treatment (Figure 2B). The immunocytochemistry analysis demonstrated a progressive reduction in immunopositivity for NRG1 protein in SCs after 24 h and 48 h from APE treatment (Figure 2C).

### 3.3. M2 Stimulation Alters erbB2 Receptor Expression and Distribution

**Figure 3:**
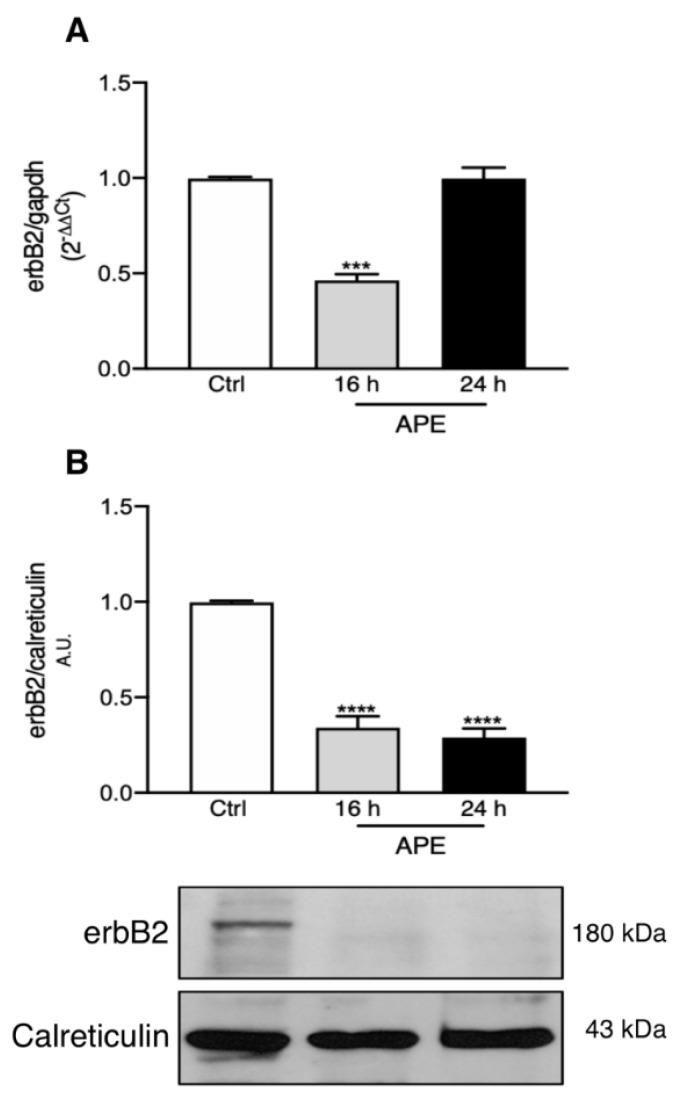
ErbB2 expression is modulated by M2 agonist APE. (**A**) qRT-PCR analysis shows the decreased expression of erbB2 receptor transcripts after 16 h of APE treatment (APE 16 h: *** *p* < 0.001; *n* = 3); (**B**) Representative Western blotting showing the decreased expression of erbB2 receptor after 16 and 24 h of APE treatment; the graph represents the average of the OD ± SEM of the bands normalized against the reference protein (**** *p* < 0.0001; *n* = 3). Calreticulin was used as reference protein considering the prevalent accumulation of erbB2 receptors in endoplasmic reticulum (ER) after APE treatment.

To respond to NRG signals, Schwann cells express erbB2 and erb3 receptors [6,31]. In order to better understand the ability of the M2 receptor to impair the NRG pathway, the expression of erbB receptors was also evaluated. As shown in Figure 3A, erbB2 receptor transcripts were significantly downregulated already after 16 h of APE treatment; after 24 h, the transcript level was comparable to untreated cells (Figure 3A). On the other hand, Western blotting analyses confirmed that erbB2 receptors were significantly downregulated after 16 and 24 h of APE treatment (Figure 3B).

**Figure 4:**
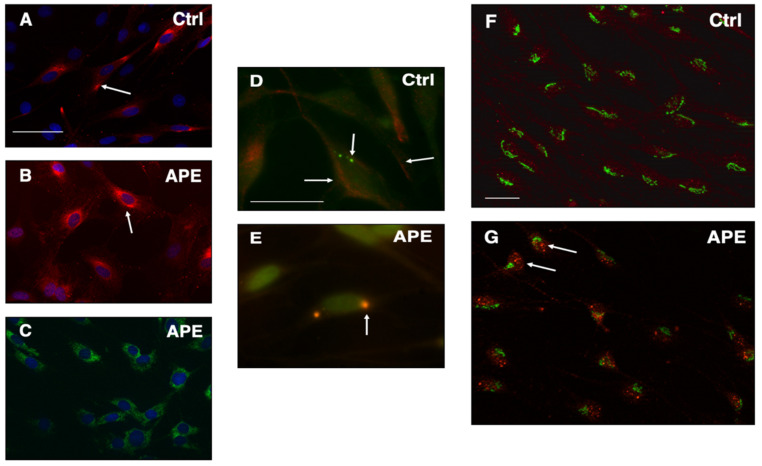
Immunocytochemistry analysis of ErbB2 expression upon M2 agonist treatment. Immunostaining for erbB2 receptors (red staining) in untreated (**A**) and APE treated SCs (**B**). The arrows indicate the different localization of erbB2 receptors. In (**C**) is shown the immunostaining for BIP (green staining), a typical ER marker (scale bar = 150 µm). The localization of erbB2 receptors changes after M2 agonist stimulation, resulting mainly in perinuclear regions of the cells. (**D**,**E**) Co-immunostaining for erbB2 (red staining) and Lamp-1 (green staining), marker of lysosome, or (**F**,**G**) GM-130 (green staining), marker of Golgi apparatus. The immunostaining clearly demonstrates that erbB2 receptors co-localize with Lamp-1, (E; scale bar = 50 µm), but not with the Golgi marker, after M2 agonist treatment (**G**), (scale bar = 100 µm). The arrows in G indicate the red spots expressing erbB2 receptors in APE treated cells that may be lysosomes/endosomes.

The immunocytochemistry analysis had also shown that the erbB2 receptor expression was mainly localized on the cell membrane surface in untreated cells (Figure 4A), whereas after APE treatment, erbB2 appeared mainly accumulated in the perinuclear area (Figure 4B). The immunolocalization of BIP protein, a typical endoplasmic reticulum (ER) protein, suggested that the perinuclear region where erbB2 receptors were localized after APE treatment may be ER region (Figure 4C). Moreover, immunostaining had even shown that erbB2 after M2 agonist treatment, co-localized with Lamp-1, marker of lysosome (Figure 4E), but not in the Golgi, as indicated by the GM-130 marker (Figure 4F–G).

### 3.4. M2 Receptor Modulates erbB2 Expression via Notch-1 Pathway

As mentioned above, Notch-1 signalling is one of the master pathways involved in the regulation of transition from precursor to immature SCs [32]. In our previous paper we demonstrated that the M2 agonist APE did not modulate the expression of full-length Notch-1 but progressively decreased the expression of the active form of Notch (Notch Intracellular Domain; NICD) [29].

**Figure 5:**
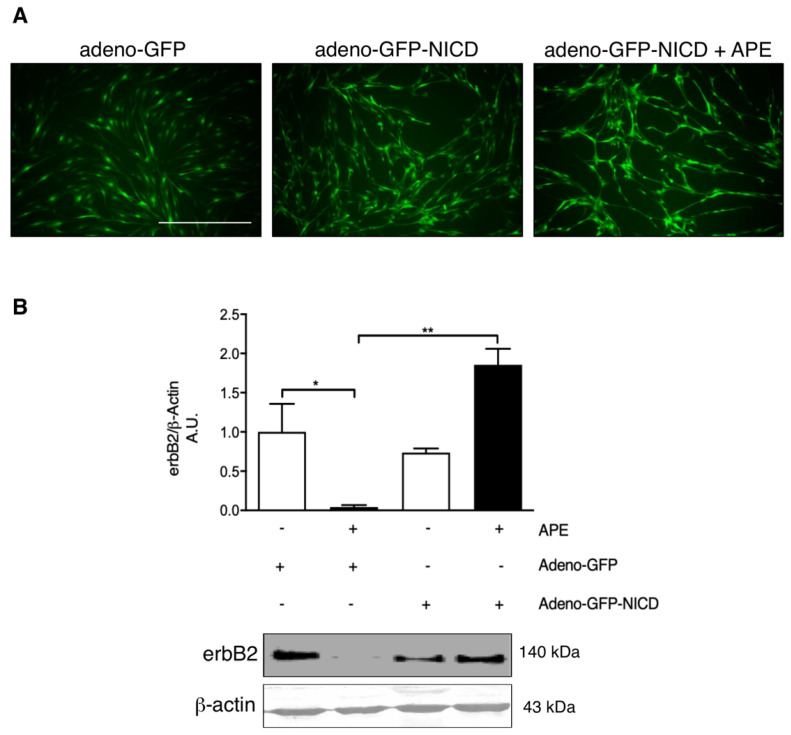
Analysis of erbB2 expression in SCs infected with Notch-NICD adenovirus in absence and in presence of M2 agonist treatment. (**A**) Representative fluorescence microscope images showing adenovirus-infected SCs expressing the adenovirus-GFP or adenovirus-NICD-GFP-constructs in control conditions and after 24 h of 100 µM APE treatment. In all pictures, SCs present the characteristic spindle-like cell morphology and a high percentage of infection (scale bar = 200 µm). (**B**) Densitometric analysis of the bands of erbB2 expressed in SCs infected with either the adenovirus-GFP or the adenovirus NICD-GFP constructs and subsequently treated with 100 µM APE when required from the experimental plan. β-actin was used as the internal reference protein. Data represent the average ± SEM of at least three independent experiments (* *p* < 0.05, ** *p* < 0.01; *n* = 3). Below the graph, representative Western blot analysis for erbB2 in SCs after infection with adenovirus-GFP or adenovirus-NICD-GFP, in the absence or presence of 100 µM APE for 24 h.

In order to evaluate whether the decreased expression of erbB2 was controlled by M2 receptors directly or via the Notch-1 pathway, infection with recombinant adenovirus expressing the construct GFP-NICD was performed. The expression of the GFP signal indicated the percentage of cells infected (Figure 5A).

In the cells infected with the control vector adeno-GFP, APE treatment induced a reduction in erbB2 protein expression, similar to that observed in not infected cells (Figure 3B and Figure 5B). On the contrary, when SCs were infected with the adeno-NICD-GFP, any variation in erbB2 protein expression was observed after 24 h of APE treatment (Figure 5B), suggesting that the erbB2 expression was directly controlled by the NICD fragment.

### 3.5. M2 Receptor Activation Negatively Controls the Expression of erbB3 Receptor

**Figure 6:**
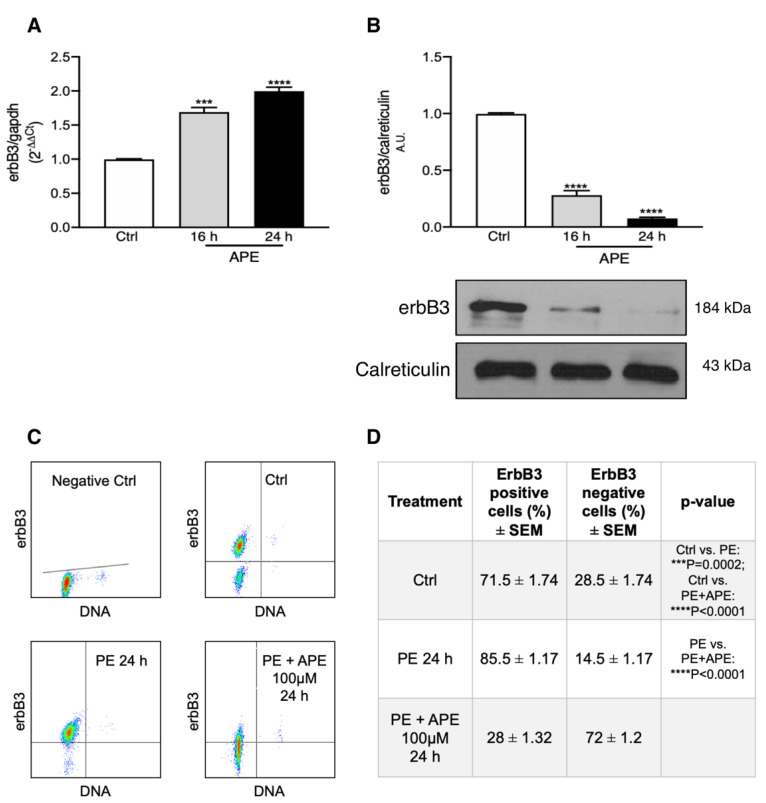
ErbB3 receptor expression in SCs. (**A**) qRT-PCR analysis shows that erbB3 receptor transcript levels are upregulated already after 16 h and 24 h of APE treatment (*** *p* < 0.001; **** *p* < 0.0001; *n* = 3); (**B**) Representative Western blot analysis for erbB3 receptor. The expression of the receptor is significantly downregulated after 16 and 24 h of 100 µM APE treatment. The graph shows the average ± SEM of the OD of the bands normalized against the OD of the calreticulin, used as reference protein (Figure 3B; **** *p* < 0.0001; *n* = 3). (**C**,**D**) FACS analysis for erbB3 receptor shows a significant increase in the percentage of erbB3 positive cells after PE treatment (85.5 ± 1.17; Ctrl vs. PE *** *p* = 0.0002; *n* = 3) and a decrease in erbB3 positive cells after APE treatment (28 ± 1.32; PE vs. PE + APE, **** *p* < 0.0001; *n* = 3).

The effect of M2 receptor stimulation on erbB3 receptor expression was also evaluated. As shown in Figure 6A, erbB3 receptor transcript levels were significantly upregulated already after 16 h and up to 24 h of APE exposure, but its protein level was significantly downregulated after 16 and 24 h of APE treatment (Figure 6B). FACS analysis for erbB3 receptor, reported in Figure 6C,D, confirmed a significant increase in the erbB3 positive cells after PE treatment (Figure 6D, 85.5 ± 1.7%), whereas the number of positive cells significantly decreased when APE was added to the SC cultures (Figure 6D, 28 ± 1.32%).

## 4. Discussion

The role of neurotransmitters in the control of neuron-glia interactions and in the modulation of glial cell development and physiology is increasingly emerging [16,20,29,33,34]. ACh, released along cholinergic axons [35], contributes to the regulation of SC proliferation to address rat and human SC differentiation towards a myelinating phenotype, increasing the expression of the transcription factor Egr2/Krox20 and myelin proteins (i.e., MBP and P0) [20,23,29].

In this work, we explored whether the effects produced by M2 receptor may be correlated with the NRG1/erbB2-3 pathway, considering the role of this growth factor in the control of SC proliferation and differentiation [6,10,31,36,37].

The FACS analysis and the MTT assay performed in the presence of PE or NRG1/I have confirmed the role of GGF/NRG1 in the positive control of SC proliferation. However, when the M2 agonist APE was added to the SC culture medium, the SC proliferative rate showed a significant reduction, indicating the ability of the M2 agonist to counteract the GGF/NRG1 effects also when the exogenous NRG or PE were provided. Moreover, when the PE was supplied to the cells, the SC ability to synthesize NRG was dramatically reduced. It is relevant to note that GGF in the pituitary extract is NRG1 type II; it may play the same regulatory role as NRG1 type III via its EGF domain in the downregulating autocrine secretion of NRG1 type I in SCs. Similarly, it appears relevant that M2 agonist APE is also able to reduce the NRG autocrine production, downregulating the NRG1 expression in SCs, both as transcript and protein.

The MTT results, however, suggest that SCs, after APE treatment, are not able to respond to NRG1 exposure, implying a possible effect of M2 agonist also on NRG receptors. The data obtained clearly demonstrate the ability of APE to negatively modulate the expression of both erbB2 and erbB3 protein expression. In particular, the erbB2 receptor transcript levels were drastically reduced after 16 h of treatment. Conversely, APE stimulation produced a progressive increase in the erbB3 and erbB2 receptor transcripts after 24 h of expression. This may suggest that the decreased expression of erbB proteins may be also due to post-transcriptional control-M2 receptor mediated, possibly regulated by some miRNA activation. However, it is not possible to exclude that the increased levels of erbB3 or erbB2 transcripts may be a compensatory effect due to the decreased expression of the respective proteins.

Immunocytochemistry analysis has shown an increased expression of erbB2 at the level of the perinuclear region after M2 stimulation, compared to untreated cells. BIP staining, a common ER marker, has shown a positivity for this marker on the same area, where erbB2 localization has been observed after M2 agonist treatment. Moreover, an increased erbB2 localization at the level of the lysosomes, as detectable by the Lamp1 co-immunostaining. Although further experiments are needed to fully address the ErbB2 localization after M2 receptor stimulation, we could assume that M2 receptor activation causes a significant decreased expression of the receptor and alters its localization on the plasma-membrane. In all cases, according to the previous observations, it is clear that after M2 receptor stimulation, SCs lose the ability to respond to NRG, with a consequent reduction in SC proliferation.

Several papers described how, in the PNS, Notch-Neuregulin1 pathways collaborate to SC survival and proliferation. Notch-1 promotes SC transition from precursors to an immature SC phenotype [32], but it also works as a negative regulator of myelination [36]. As a matter of fact, overexpression of the active intracellular domain of Notch1 (NICD) delays myelination, while Notch-1 inactivation accelerates it [32,38]. In our previous work, the analysis of Notch-1 and the active form NICD expression in cultured SCs after APE treatment showed that, although the expression of full-length Notch-1 was not modulated, NICD was progressively reduced in APE-treated cells in a dose dependent manner [29]. Considering that the M2 agonist is able to downregulate the expression of the erbB2 receptor at a transcriptional and protein level, we tried to understand if these two effects were related to each other. The infection of SCs with adenovirus expressing NICD-GFP has allowed us to demonstrate that M2 agonist treatment was not able to downregulate erbB2 expression in NICD-GFP infected cells, whereas a significant downregulation was observed in not infected SCs and in SCs infected with adenovirus containing an GFP-empty construct. This result suggests that the levels of erB2 are indirectly controlled by M2 selective activation, through NICD downregulation.

## 5. Conclusions

M2 muscarinic receptor activation, through the selective agonist APE, is able to decrease the expression of erbB receptors on the plasma-membrane of the SCs, preventing NRG1 binding. This result, supported by immunocytochemistry and FACS analysis studies, suggests that the M2 receptor is able to alter the formation of the erbB2/3 complex. Moreover, a cross-interaction between M2 receptor-Notch-1 (NICD) and NRG/erbB pathways was also defined; in fact, M2 receptor activation, downregulating Notch active form NICD, indirectly inhibits the erbB2 receptor expression.

These data, in agreement with the results obtained from our previous studies [16,20,23,29], suggest that the cholinergic stimulus mediated by the M2 muscarinic receptor, may be used by the SCs as a signal to counteract cell proliferation by switching off the Notch-NRG pathways.
